# Psychosocial burden and professional and social support in patients with hereditary transthyretin amyloidosis (ATTRv) and their relatives in Italy

**DOI:** 10.1186/s13023-021-01812-6

**Published:** 2021-04-07

**Authors:** Lorenza Magliano, Laura Obici, Claudia Sforzini, Anna Mazzeo, Massimo Russo, Francesco Cappelli, Silvia Fenu, Marco Luigetti, Matteo Tagliapietra, Chiara Gemelli, Luca Leonardi, Stefano Tozza, Luca Guglielmo Pradotto, Giulia Citarelli, Alessandro Mauro, Fiore Manganelli, Giovanni Antonini, Marina Grandis, Gian Maria Fabrizi, Mario Sabatelli, Davide Pareyson, Federico Perfetto, Giampaolo Merlini, Giuseppe Vita, ATTRv Collaborators, Giulia Bisogni, Daniela Calabrese, Davide Cardellini, Silvia Casagrande, Tiziana Cavallaro, Eleonora Di Buduo, Andrea Di Paolantonio, Luca Gentile, Anita Graceffa, Sara Massucco, Alessandra Milesi, Stefania Morino, Roberta Mussinelli, Paola Saveri, Daniele Severi

**Affiliations:** 1 Department of Psychology, University of Campania “Luigi Vanvitelli”, Viale Ellittico 31, 81100 Caserta, Italy; 2 Amyloidosis Research and Treatment Center, IRCCS Fondazione Policlinico San Matteo, University of Pavia, Pavia, Italy; 3 Department of Clinical and Experimental Medicine, University of Messina, Messina, Italy; 4 Tuscan Regional Amyloidosis Centre, Careggi University Hospital, Florence, Italy; 5 Rare Neurodegenerative and Neurometabolic Diseases Unit, Department of Clinical Neurosciences, Fondazione IRCCS Istituto Neurologico Carlo Besta, Milan, Italy; 6 Fondazione Policlinico Universitario A. Gemelli IRCCS. UOC Neurologia, Rome, Italy; 7 Dipartimento Di Neuroscienze, Università Cattolica del Sacro Cuore, Rome, Italy; 8 Department of Neuroscience, Biomedicine and Movement Sciences, University and AOUI Verona, Verona, Italy; 9 Department of Neurosciences, Rehabilitation, Ophthalmology, Genetic and Maternal and Infantile Sciences (DINOGMI), University of Genoa, Genoa, Italy; 10 IRCCS Ospedale Policlinico San Martino, Genova, Italy; 11 Department of Neurosciences, Mental Health and Sensory Organs, Faculty of Medicine and Psychology, Sant’Andrea Hospital, Sapienza University of Rome, Rome, Italy; 12 Department of Neurosciences, Reproductive Sciences and Odontostomatology, University of Naples, Federico II, Naples, Italy; 13 Department of Neuroscience “Rita Levi Montalcini”, University of Turin, Turin, Italy; 14 Unit of Neurology and Neurorehabilitation, IRCCS - Istituto Auxologico Italiano, Piancavallo, VB Italy; 15 NEuroMuscular Omnicentre (NEMO), Fondazione Policlinico Universitario A. Gemelli, IRCCS, Rome, Italy

**Keywords:** Hereditary transthyretin amyloidosis, ATTRv, Burden, Professional support, Social network support, Caregiving

## Abstract

**Background:**

Hereditary transthyretin amyloidosis (hATTR), alias ATTR variant (ATTRv) is a severe and disabling disease causing sensory and motor neuropathy, autonomic dysfunction, and cardiomyopathy. The progressive decline of patient’s functional autonomy negatively affects the patient’s quality of life and requires increasing involvement of relatives in the patient’s daily life. Family caregiving may become particularly demanding when the patient is no longer able to move independently. This study is focused on the psychosocial aspects of ATTRv from the patient and relative perspectives. In particular, it explored: the practical and psychological burdens experienced by symptomatic patients with ATTRv and their key relatives and the professional and social network support they may rely on; whether burden varied in relation to patients’ and relatives’ socio-demographic variables, patients’ clinical variables, and perceived professional and social network support; and, any difference in burden and support between patients and their matched relatives.

**Methods:**

The study was carried out on symptomatic patients included in the ATTRv Italian national registry and living with at least one adult relative not suffering from severe illness and being free from ATTRv symptoms. Patients and relatives’ assessments were performed using validated self-reported tools.

**Results:**

Overall, 141 patients and 69 relatives were evaluated. Constraints of leisure activities, feelings of loss and worries for the future were the consequences of ATTRv most frequently reported by patients and relatives. Both in patients and their relatives, the burden increased with the duration of symptoms and the level of help in daily activities needed by the patient. In the 69 matched patient-relative pairs, the practical burden was significantly higher among the patients than among their relatives, while the psychological burden was similar in the two groups. Moreover, compared to their relatives, patients with ATTRv reported higher levels of professional and social network support.

**Conclusions:**

These results show that ATTRv is a disease affecting quality of life of both patients and their families. Supporting interventions should be guaranteed to patients, to facilitate their adaptation to the disease, and to their families, to cope as best as possible with the difficulties that this pathology may involve.

**Supplementary Information:**

The online version contains supplementary material available at 10.1186/s13023-021-01812-6.

## Background

Hereditary transthyretin amyloidosis (hATTR), alias ATTR variant (ATTRv) is a severe and disabling disease causing sensory and motor neuropathy, autonomic dysfunction, and cardiomyopathy [1]. This disease is present worldwide, with the highest prevalence in Northern Portugal, Northern Sweden, Japan, Majorca and Cyprus [2–5]. ATTRv has an autosomal dominant transmission due to missense mutations in the TTR gene, located on chromosome 18. Mutated protein monomers aggregate to form amyloid fibrils, which precipitate in different tissues causing dysfunction, such as peripheral nerves, heart, gastrointestinal tract, kidney, and eye. Average survival from disease onset varies from 6 to 12 years and cardiac involvement is often the cause of death [6]. The disease is classified on the basis of age at onset, and symptoms before the age of 50 distinguish early from late onset ATTRv [6]. The course varies in relation to the type of mutation and age of onset. Late-onset patients with common mutation Val30Met (Table 1) from non-endemic areas have different phenotype from early-onset cases from endemic foci in Portugal and Japan, since these patients tend to manifest loss of all sensory modalities, mild autonomic dysfunctions, and heart failure with massive cardiac amyloid deposition. If patients are untreated, late-onset Val30Met patients from non-endemic areas have much shorter mean survival time than early-onset Val30Met patients from endemic foci [4, 7, 8]. Symptomatic treatment requires a multidisciplinary approach addressing heart manifestations, orthostatic hypotension, gastrointestinal disorders with weight loss, and neuropathic pain. For two decades, liver transplantation has been the only treatment for halting or slowing amyloid deposition in target organs. Since 2011, tafamidis meglumine has been used to slow the progression of the disease in neuropathic patients. More recently, two innovative and very effective gene silencing therapies have reached the market [9].

**Table 1:** Patients and relatives socio-demographic characteristics and patients’ clinical variables

	Patients (N = 141)		Relatives (N = 69)	
	N	%	N	%
Socio-demographic characteristics				
Gender				
Males	106	75.2	15	21.7
Females	35	24.8	54	78.3
Age, mean ± SD	66.1 ± 11.09		57.2 ± 11.8	
Marital status				
Free	34	25.2	9	10.5
Married	101	74.8	60	89.6
Education				
Primary school	18	14.3	10	14.7
Secondary school	42	33.3	14	20.6
High school	44	34.9	32	47.1
University	22	17.5	12	17.7
Employed, yes	41	29.5	32	47.1
Relationship with the patient	–			
Spouse			51	75.0
Other			17	25.0
Daily hours spent in caregiving, mean ± SD	–		8.1 ± 9.7	
Clinical variables				
Mutation				
Val30Met	39	27.6		
Phe64Leu	33	23.4		
Glu89Gln	25	17.7		
Ile68Leu	14	9.9		
Val122Ile	7	5		
Tyr78Phe	7	5		
Thr49Ala	6	4.3		
Other (n. 9)	10	7.1		
Phenotype			–	
ATTRv-PN	111	78.7		
ATTRv-CM	30	21.3		
ATTRv stage			–	
0	6	4.3		
1	75	53.2		
2	42	29.8		
3	18	12.8		
Age at symptom onset, mean ± SD	59.8 ± 11.8		–	
Length of symptom in years, mean ± SD	6.2 ± 4.2		–	

Mutation nomenclature follows the traditional classification which refers to the TTR protein (NCBI Reference Sequence: NP_000362.1; chromosome 18, NC_000018.10) without the first 20 amino acids of the leader peptide. For example, Val30Met corresponds to p.(Val50Met) if one follows the Human Genome Variation Society (HGVS) nomenclature (https://www.hgvs.org/content/guidelines)

The progressive decline of neurological functions, which is variably associated with disabling cardiological and dysautonomic symptoms, negatively affects the patient’s quality of life and requires increasing involvement of relatives in the patient’s daily life. Family caregiving may become particularly demanding when the patient is no longer able to move independently. In the late stages of the disease full-time assistance and hospitalization may be necessary. While burden of care has been extensively explored in mental and neurological diseases [10, 11], few studies have specifically investigated the practical and psychosocial difficulties experienced by patients with ATTRv and their families. Available data reveal that patients with this disease have difficulties in marital relationships and social life and fear of losing their functional autonomy and becoming dependent on family for help [12–14]. Worsening symptoms and disease-related disability often led to inability to work, and they contributed to feelings of frustration and sadness [14]. Furthermore, these patients sometimes perceive to be discriminated against and negatively judged by others if they plan to have children [15]. Moreover, both patients with ATTRv and their families are more vulnerable to emotional stress and psychopathology during their lifetimes [16, 17], and they have poorer quality of life than the general population [18–20]. In at-risk relatives, significant long-term psychosocial burden and fear of death have also been reported [17, 20, 21]. These studies have several weaknesses limiting the generalizability of their results. Some studies have small sample sizes and were conducted using a qualitative design [14, 20, 21], a methodology which is difficult to apply in routine clinical settings. Furthermore, only a few studies have examined the burden of care both from the patient’s and the relative’s perspective [12, 18, 20], and none has specifically examined the impact of professional or social support on the patient’s or relative’s burden. The lack of representative data on the burden in ATTRv makes it more difficult to develop ad hoc supportive interventions for patients and their families.

In the period from 2016-2019, within the framework of the Telethon National Program for clinical research, a study on ATTRv was carried out in Italy. The study aimed to create a national registry collecting clinical, treatment-related, and genetic information of the disease [22, 23]. Moreover, the study investigated the burden of care and the professionals and social support in a large sample of symptomatic patients and their key-relatives, using validated, self-reported questionnaires. To our knowledge, this is the first study including both patients and relatives’ psychosocial data carried out at national level.

This paper is focused on the psychosocial aspects of ATTRv from the patient and relative perspectives. In particular, we explored: (a–b) the practical and psychological burdens experienced by symptomatic patients with ATTRv and their key relatives and the professional and social network support they may rely on; (c) whether burden varied in relation to patients’ and relatives’ socio-demographic variables, patients’ clinical variables, and perceived professional and social network support; d) any difference in burden and support between patients and their matched relatives.

## Methods

### Study design and procedures

The study was carried out in ten clinical centres located throughout Italy (five in Northern, three in Central and two in Southern Italy). All centres were multidisciplinary, but eight were coordinated by a neurologist, one by a cardiologist and one by an internist. An informatics technology platform dedicated to the ATTRv Registry was developed and became effective in 2017. In the period from June 2017-November 2019, patients and their key relatives (i.e., the relative most involved in the patient’s daily assistance) who met the below-mentioned selection criteria were contacted consecutively and invited to participate in the study. Patient selection criteria were the following: being included in the ATTRv National Registry; being symptomatic for ATTRv; living with at least one adult relative. There were no age restrictions. Key-relative selection criteria were the following: age between 18 and 80 years; not suffering from illness requiring long-term intensive care; being free from ATTRv symptoms.

During a scheduled follow-up visit, each eligible patient who gave written informed consent to participate in the study was invited to complete: (a) the patient version of the Problems Questionnaire (PPQ) [24, 25]; (b) the Social Network Questionnaire (SNQ) [25, 26]; (c) the ATTRv non-pharmacological Patient Care Schedule (ATTRv-PCS). At the same occasion, each eligible key relative who agreed to participate and signed an informed consent was asked to complete: (a) an ad-hoc family socio-demographic schedule; (b) the family version of the Problems Questionnaire (FPQ); (c) SNQ (data not reported in this paper); (d) the ATTRv-Family Support Schedule (ATTRv-FSS). Patient and relative socio-demographic characteristics were collected by ad hoc schedules. The above-mentioned instruments were self-administered in the presence of a trained researcher. Participants did not receive any financial or other incentives for their participation in the study. Furthermore, information on current phenotype (predominantly PolyNeuropathy, ATTRv-PN; predominantly CardioMyopathy, ATTRv-CM), type of mutation, age at the onset of symptoms, and ATTRv stage (stage 0, subjects asymptomatic for neuropathy, but showing only cardiomyopathy; stage 1, symptomatic neuropathy with unimpaired walking; stage 2, need of mono- or bilateral walking assistance; stage 3, patient confined to a wheelchair [27] was drawn from the ATTRv National Registry.

The study protocol was approved by the Ethics Committee of the University of Messina (coordinating centre), and by the Local Ethics Committee of each participating centre. The study was performed in accordance to the ethical standards laid down in the 1964 Declaration of Helsinki and its later amendments.

### Instruments descriptions

Problems Questionnaire: the patient-version PPQ contains the same items as the family-version FPQ but reformulated to refer to the patient’s first-person experience. The tool explores: (a) the help needed by the patient in performing daily activities and adhering to care (5 items); (b–c) the support provided by health professionals (5 items) and their social network in clinical emergencies (2 items); (d–e) the practical and psychological burden of care (9 items and 7 items, respectively). Items are rated on a 4-level scale from 1 “never” to 4 “always” (sections d–e) and from 1 “not at all” to 4 “completely” (remaining sections). The reference period is the last 2 months except for sections *b–c* which do not include a specific reference period. An average subscale’ score is derived across the valid scores of the items included in each subscale. The questionnaire also contains additional sections on the economic costs of care, the respondent’s perceptions of the illness as a condition also having positive aspects, and an open-ended question where respondents can offer suggestions to improve the condition of patients with ATTRv and their relatives. Cronbach’s alphas values, measured in this study samples, were acceptable (*a* to *e* subscales: from .70 to .92 in the PPQ and from .65 to .93 in the FPQ).

Social Network Questionnaire: the SNQ is a self-reported tool exploring the respondent’s: (a) quantity and frequency of social contacts (4 items); (b–d) perception of practical and psychological support received from social network and the partner (3 items, 4 items, and 2 items, respectively). Items are rated on a four-level scale, from 1 “Never” to 4 “Always”. In this study, Cronbach alphas of the SNQ subscales ranged from .65 to .73 in the patient sample and from.66 to .81 in the relative sample. Both the PPQ/FPQ and the SNQ were administered in their entirety, but, as this study is based on PPQ/FPQ section *a–e* and the suggestions section, SNQ items were not reported here.

ATTRv-non-pharmacological Patient Care Schedule (ATTRv-PCS): this schedule contains seven “Yes/No” items on rehabilitative treatments, professional support, self-help and association support, and economic benefits received by the patient in the previous 12 months.

ATTRv-Family Support Schedule (ATTRv-FSS): this schedule contains five “Yes/No” items on the support that key relatives have received from professionals, self-help groups, and associations in the previous 12 months.

### Statistical analysis

Frequencies and percentages were calculated for categorical and ordinal variables describing: socio-demographic characteristics of patients and their key relatives (sex, marital status, level of education, current employment, relationship with the patient); patients’ clinical features (type and stage of ATTRv); help needed by the patient in daily activities and care (PPQ and FPQ *a* subscale items); burden (PPQ and FPQ *d-e* subscales items); professional and social support (PPQ and FPQ *b-c* subscales items). Means and standard deviations (SD) were computed for continuous variables as patients’ and key-relatives’ ages, relatives’ daily hours dedicated to patient’s care, patients’ length of illness, and for burden and support subscale scores (PPQ and FPQ *a-e* sections).

In each group (patient group and relative group), Spearman’s r coefficient was computed to investigate the relationships of practical and psychological burden (PPQ/FPQ *d-e* subscales, respectively) with patients’ and relatives’ age, patients’ duration of illness, level of help needed in daily activities (PPQ/FPQ subscale *a* mean score, respectively), and professional and social network support (PPQ/FPQ *b-d* subscales mean scores, respectively). ANOVA test was performed to explore the associations of burden with respondent’s sex, patient’s type of ATTRv, and supportive interventions (ATTRv-PCS and ATTRv-FSS items). T-test for paired data was used to investigate differences in burden and professional and social network support among patients and their participating relatives. Statistical significance was set at *p* < 0.05. Analyses were performed by using SPSS 21.0.

## Results

### Sample characteristics

**Fig. 1:**
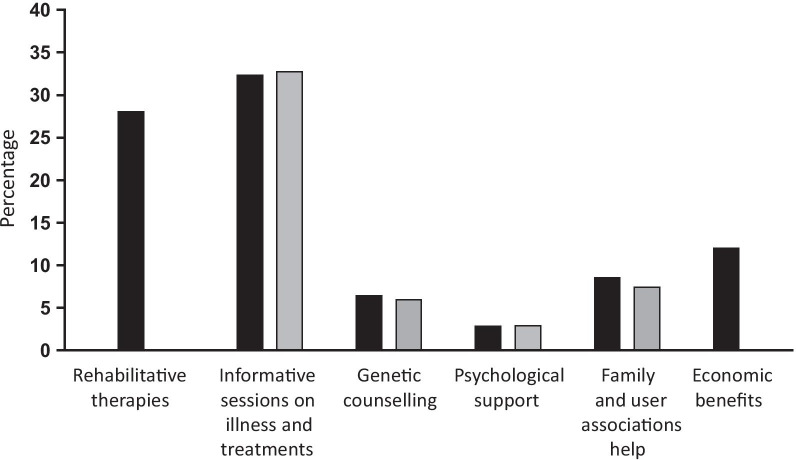
Non-pharmacological interventions and support received by ATTRv patients and their relatives (N:69) in the past year. Black square, patients; grey square, key-relatives

Of the 143 eligible patients and their key-relatives who were consecutively contacted, 141 patients and 69 key-relatives agreed to participate in the study and were assessed (refusal rates: 0.01% and 51%, respectively. Patients’ refusal reasons: 2 unknowns. Relatives’ refusal reasons: 25 unavailable to accompany the patient to the clinical examination, 9 lack of interest in the study, 38 unknown). The 69 patients whose key-relatives participated were comparable with the 72 patients whose key-relatives did not with respect to the main socio-demographic variables (sex, age, occupation) except for marital status (60/69 married patients among those whose relatives participated vs. 42/66 married patients among those whose relatives were not assessed, χ^2^ = 8.6, df 1, *p* < .005), and with respect to the main clinical characteristics (duration of symptoms, phenotype and FAP stage) except for help needed in daily activities (mean score ± sd: 2.0 ± 1.0 vs. 1.6 ± 1.1, t = 2.1, df 139, *p* < .03). The 141 participating patients were on average 66.1 (11.09 SD) years old and symptomatic on average for 6.2 (4.2 SD) years (Table 1). One hundred and eleven (78.7%) patients suffered from ATTRv-PN, and 30 (21.3%) from ATTRv-CM. Fifty-three percent of the patients had a predominantly sensory neuropathy affecting their lower limbs and walked without any help (stage 1: n. 75); 29.8% needed help walking (stage 2: n. 42), and 12.8% were in wheelchairs (stage 3: n. 18). Fifty percent of patients said they needed help to get around and 44.7% needed help to visit their doctor and take medications in the previous 2 months (see Additional file 1: Table S1). Twenty-eight percent of patients attended rehabilitation programs, 32.4% had attended informative sessions on ATTRv, 2.9% had received psychological support, and 12.1% had received economic benefits (Fig. 1).

Of the 69 participating key relatives, most were female and spouses (Table 1), highly educated and not employed. Relatives stated that they spent on average 8.1 (9.7 SD) daily hours in patient caregiving over the previous 2 months (note: 12 relatives stated that they were 24-hour/day involved in the patient’s assistance). In particular, 60.9% of the relatives stated that in the previous 2 months the patient had needed their help to make medical visits or take medication, and 59.4% stated that the patient needed their help to get around (see Additional file 1: Table S1). Thirty-three percent of relatives had attended informative sessions on ATTRv, 3.0% had received psychological support and 7.5% had received help from family/patient associations (Fig. 1).

### Practical and psychological burden and perception of professional and social network support in patients with ATTRv and their relatives

The practical consequences of ATTRv most frequently reported by patients with respect to the previous 2 months concerned the neglect of hobbies and leisure time activities (72.9 % of patients) and awakening during the night (69.1%). Sixty-seven percent of patients stated that they had difficulties in carrying out usual work or household activities due to their own health condition. As far as the psychological burden, 80.6% of patients felt worried for the future of other family members (see Additional file 1: Table S2), 77.7% had feelings of loss and 66.0% thought that if there had no family members, no one would have taken care of him/her. Sixty percent of patients stated they had cried or felt depressed in the past 2 months. As far as the support that patients received from professionals and their social networks (see Additional file 1: Table S3), most patients reported receiving adequate information from their clinicians on ATTRv (87.9%) and its treatments (85.6%). Moreover, 44.3% were completely confident they would receive professional help in a crisis situation. Sixty-nine percent of patients were sure that their friends and relatives would definitely help them in the case of clinical emergencies.

Regarding the condition of the 69 participating relatives, 60.9% stated that they had to neglect their hobbies due to the patient’s condition, 60.7% had difficulties in going on Sunday outings and 59.3% in going for holidays (see Additional file 1: Table S4). Seventy-eight percent of relatives had feelings of loss, and 75.0% felt worried for the future of other family members. Sixty-four percent of relatives thought the family was the only source of help available to the patient. Fifty-nine percent of relatives stated they had cried or felt depressed due to the patient’s condition in the past 2 months. Thirty-eight percent of relatives reported they were sure they would receive professional help in an emergency concerning the patient, and the majority of relatives believed that they had received adequate information from clinicians on the disease and its treatments (72.1% and 77.9%, respectively). Forty-six percent of the relatives felt their friends would definitely help them in the event of patient’s emergencies (see Additional file 1: Table S5).

### Relationships of burden with socio-demographic and clinical variables and professional and social support in patients and relatives’ groups

In the sample of 141 patients, the practical and psychological burden increased in relation to the patient’s level of help needed in daily activities and weakly in relation to the duration of symptoms (Table 2). The practical burden was higher among patients with more severe ATTRv stages and the psychological burden was higher among patients with ATTRv-PN vs. ATTRv-CM. The burden was higher among patients receiving rehabilitative interventions and among patients who had attended informative sessions on the disease in the last year.

**Table 2:** Relationships of burden with socio-demographic and clinical variables and professional and social support

	Practical burden	Psychological burden
Patients group		
Help to patient in daily activities and care, *r value*	.63^d^	.54^d^
Duration of symptoms, *r value*	.26^c^	.22^b^
ATTRv stage, mean (sd)		
0	1.4 (0.5)	–
1	2.0 (0.7)	–
2	2.2 (0.4)	–
3	2.3 (0.7)	–
*F*, *df*	3.5; 3, 137 ^a^	–
ATTRv type, mean (sd)		
PN	–	2.1 (0.7)
CM	–	1.8 (0.5)
*F*, *df*	–	5.2; 1,139^a^
Rehabilitative treatments, mean (sd)		
Yes	2.6 (0.6)	2.5 (0.6)
No	1.9 (0.7)	1.9 (0.6)
*F*, *df*	26.1; 1, 137^d^	34.0; 1,137^d^
Informative sessions on the disease, mean (sd)		
Yes	2.3 (0.7)	2.3 (0.7)
No	1.9 (0.7)	1.9 (0.6)
*F*, *df*	5.9; 1,134^a^	7.03; 1,134^b^
Relatives group		
Social network support in emergencies, *r value*	− .27^a^	− .34^c^
Help to patient in daily activities and care, *r value*	.65^d^	.58^d^
Duration of symptoms, r value	66^d^	.58^d^
Relatives’ sex, mean (sd)		
Male	–	1.7 (0.6)
Female	–	2.1 (0.7)
*F*, *df*	–	5.1; 1, 67^a^
ATTRv type, mean (sd)		
PN	–	2.1 (0.7)
CM	–	1.5 (0.5)
*F*, *df*	–	7.2; 1, 67^b^

^a^*p* < .05; ^b^*p* < .01; ^c^*p* < .005; ^d^*p* < .0001

In the sample of 69 relatives, the practical and psychological burden were higher in relatives with lower social support for patient’s emergencies, in relatives who were more involved in providing daily help to the patient, and weakly in relatives of patients with a longer duration of symptomatic ATTRv (Table 2). Furthermore, the psychological burden was higher among female vs. male relatives, and among relatives of patients with ATTRv-PN vs. ATTRv-CM.

### Paired sample comparisons of burden and support

**Fig. 2:**
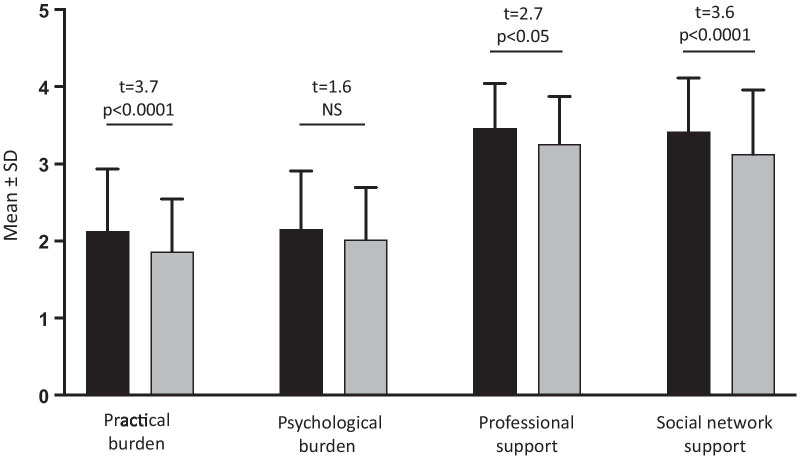
Paired sample comparisons of burden and support in patients with ATTRv and their relatives (N:69). Black square, patients; grey square, key-relatives. Paired sample t-test. NS, not significant

The comparisons of burden and support between the 69 matched patient-relative pairs are reported in Fig. 2. The analyses revealed that the practical burden was significantly higher among the patients than among their key relatives, while the psychological burden was similar in the two groups. Moreover, compared to their relatives, patients with ATTRv reported higher levels of professional and social network support.

### Participants’ recommendations on how to improve the condition of patients with ATRv and their relatives

Recommendations on how to improve the conditions of patients with ATTRv and their families were given by 61 patients and 32 relatives. In particular, 26.2% of patient and 25.0% of relatives suggested to improve the quality of care (for instance, through the implementation of local health centres covering all clinical and rehabilitative needs of patients with ATTRv, and the provision of at-home treatments and practical support). Moreover, 22.9% of patients and 37.5% of relatives recommended providing more psychological support and information to patients and their families, and 34.4% outlined the importance of investing more in research into treatments. Other recommendations were: providing education on ATTRv to health professionals, particularly GPs (8.2% and 3.1%), simplifying bureaucratic procedures and providing economic support to the patients (4.9%), promoting sensitization initiatives about ATTRv for the public (1.6% and 3.1%), and setting up self-help groups throughout Italy (1.6% and 3.1%).

## Discussion

This is the first study carried out in Italy that has systematically explored the difficulties experienced by patients with ATTRv and their families as well as the professional and social resources on which patients and families may rely. The large sample size of patients,  recruited in 10 clinical centers located in different geographical areas of Italy,  makes these data representative of the national situation and useful for comparisons with the burden in other countries and in other rare diseases. These data could be also useful in the development of ad-hoc supportive interventions for patients with ATTRv and their families. Moreover, since the patients are included in the national ATTRv Registry, it will be possible to conduct follow-up reassessment examining the burden in relation to the disease course. The replication of this study in countries with different health policies could be facilitated by the use of validated questionnaires available in different languages [25] and in patient and family versions.

The study revealed both similarities and differences in burden and in resources in patients with ATTRv and their relatives. In both groups, constraints of leisure activities are the practical consequences of the patient’s illness most frequently reported as present in the past 2 months (72.30% and 60.9%). These limitations—also found in other disabling diseases—can negatively affect the social life of the whole family [10, 11, 13]. This situation can be particularly unfavorable in elderly patients, due to the spontaneous decline of social networks in old age. It is noteworthy that only 5.8% of patients reported frequent economic difficulties due to their medical condition. This result is probably related to Italian health legislation that guarantees all citizens free access to public health services [28].

As far as the psychological consequences are concerned, worries for the future of other family members are high in both patients and relatives (81.6% and 75.0%). In addition, feeling of loss in thinking about the negative life changes that the illness has imposed on the patient are similarly reported by patients and family members (77.7% and 77.9%). These findings highlight that ATTRv has negative effects on relatives’ quality of life, a circumstance that sometimes may fuel feelings of frustration in patients [14]. The familiar hereditary influence of ATTRv is reflected in the feeling of guilt of a possibility to pass the disease to offspring, as found in the 58.2% of the patients and 39.5% of their relatives. Moreover, it is likely that feelings of loss and worries about the future—frequently reported by patients and relatives in this study—subtend fear of death, sometimes observed in patients with ATTRv and their at-risk relatives [16, 17, 20]. Sixty-six percent of patients and 48.5% of family members were convinced that if the patient had not been ill everything would have been fine in their family. This conviction further outlines the feeling of guilt in these patients. Sixty percent of patients and key relatives stated they cried or felt depressed in the past 2 months. These findings are in line with those reported by Lopes et al. [16] about the prevalence of psychiatric problems in ATTRv patients and by Theaudin et al. [12] on marital difficulties and depression in this disease. Feelings of sadness may lead to depression and other minor psychiatric disorders [18]. These findings emphasize the importance of providing psychological support to the entire family when necessary.

In both patients and key relatives, the subjective burden is higher than the objective burden. This finding is in line with previous studies on long-term diseases [10, 11] and it highlights that psychological consequences can be heavy even when the practical help needed by the patient is still limited. In this regard it should be noted that 57.5% of the patient sample was in conditions of functional autonomy (FAP stage 0-1), being able to walk without help.

Analyses on matched patient-relative pairs revealed that practical difficulties are significantly higher among patients than relatives, whereas psychological difficulties are similar in the two groups. This result highlights how the psychological consequences of the disease on key relatives can be substantial even when the practical family burden is lower than that faced by the patient. It is likely that aspects such as concern about the outcome of the disease, previous family experience of parents or siblings of the devastating effects of the disease and the meaning of ATTRv in the family relationships affect the overall level of psychological difficulties of the patient as well as his/her relative. It should also be noted that most relatives were spouses with an average age of 60.9 years (9.1 sd). It is likely that providing practical help to the patient can be a considerable physical strain for relatives of that age.

Most patients and family members had adequate information about the disease (87.9% and 72.1%) and treatment (85.6% and 77.9%) from physicians. In addition, about 35% of the sample participated in sessions dedicated to information about ATTRv. While education on the illness and treatments were guaranteed to most patients and their families, a very small percentage (2.9% and 3.0%) received psychological support from professionals. The lack of psychological support is an unmet need and was recommended among the suggestions to improve the quality of life by 22.9% of patients and 37.5% of their relatives, respectively. The lack of psychological support—common in physical and mental pathologies [10, 11, 13]—is partly explained by the lack of attention paid to the psychological aspects of care in the medical training, and the poor integration between medicine and health psychology. However, reluctance of patients and families to seek psychological support due to stigma mechanisms cannot be excluded [15]. Furthermore, the study revealed that compared to their key relatives, patients stated that they could rely on greater support from professionals and their social networks, suggesting greater solitude in caregivers than in the patients.

The study has some limitations that should be taken into account in the interpretation of the results. One limitation is that the study sample includes only symptomatic individuals. Therefore, the results of this study cannot be generalized to asymptomatic patients and their relatives that may present different levels of psychological burden related to worries about the unfavorable outcome of the disease [13]. Other limitations are the lack of information on whether the key-relative is an asymptomatic carrier and the potential commitment of participants in caregiving for other relatives in the past [17]. Moreover, the study did not examine the burden on non-key relatives that could be as high as that of the key relative [29]. Furthermore, the study included only patients living with at least one adult relative. Therefore, the study did not explore the burden in patients receiving daily assistance by formal caregivers (i.e., persons paid for providing care). This condition may influence the burden perceived by the patients (for example, it can be associated to higher sense of loneliness) as the burden on non-cohabiting family members (for example, it can be associated with lower levels of family difficulties in social and work activities and with higher economic costs). Another limitation is the lack of a control group which makes it difficult to interpret the results. An approximate comparison of burden experienced by ATTRv relatives with a previously tested sample of 120 key-relatives of adult patients in charge to specialized units for brain disorders [10] by using the same questionnaire, revealed similar levels of objective burden in the two sample (brain disorders family objective burden: mean ± sd: 2.2 ± 0.7, t = 1.15, df 259, *p* < .25) and higher levels of subjective burden in the brain disorders sample (2.4 ± 0.6, t = 3.68, df 259, *p* < .0003). Another approximate comparison of burden in relatives of patients with ATTRv with that experienced by 502 key-relatives of 4–25 years-old patients with muscular dystrophies [11] using the same questionnaire revealed statistically significant differences between the two samples. In particular, both objective and subjective burden were higher among the relatives of ATTRv patients than among the relatives of patients with muscular dystrophies (objective burden: 1.6 ± 0.6, t = 8.42, df 641, *p* < .0001; subjective burden: 1.9 ± 0.6, t = 3.37, df 641, *p* < .0008). Results from the above-mentioned comparisons suggest that burden is a complex phenomenon in which variables related to the type of pathology and its outcome interact with variables related to the relationship between patient and relative. A further limitation is that only 69 out of 141 expected key-relatives participated in the survey. The 51% refusal rate may have inflated the results on the average family burden which may in fact be lower in case of more autonomous patients who did not need to be accompanied to clinical visits (daily help needed by the non-accompanied patients, mean score ± sd: 1.6 (1.1) vs. accompanied patients: 2.0 (1.0). It is likely that the 51% refusal rate also depends on the fact that in this group more often the patients were unmarried (36.4% vs. 14.5%).

## Conclusions

Overall, the results of this study show that ATTRv is a disease affecting quality of life of both patients and their families. Supporting interventions should be guaranteed to patients, to facilitate their adaptation to the disease, and to their families, to cope as best as possible with the difficulties that this pathology may involve. Family support should include: providing step-by-step information on ATTRv and currently available treatments to patients and their informal caregivers; using a problem-solving approach to help patients and family members cope with the practical difficulties associated with the disease; and, encouraging patients and relatives to share their experience and seek mutual support, for example by joining self-help groups and patient and relative associations.

## Supplementary Information

**Additional file 1**. **Table S1**, entitled “Help in daily activities needed by the patients with ATTRv and provided by their relatives in the past two months”; **Table S2**, entitled “Practical and psychological burden experienced by patients with ATTRv (N=141) in the last two months”; **Table S3**, entitled “ Perceived support received from professionals and social network by patients with ATTRv (N=141)”; **Table S4**, entitled “Practical and psychological burden experienced by relatives of patients with ATTRv (N=69) in the last two months”; **Table S5**, entitled “Perceived support received from professionals and social network by relatives of patients with ATTRv (N=69)”.

## Data Availability

The data that support the findings of this study will be made available from the corresponding author upon reasonable request. The request must include a protocol and statistical analysis plan and not be in conflict with our publication plan. Requests for data sharing will be considered by the corresponding author (Lorenza Magliano) and the Scientific Coordinator of the GUP15010 study (Giuseppe Vita).

## Abbreviations

ATTRv: Transthyretin-related amyloidosis, variant
hATTR: Hereditary transthyretin-related amyloidosis
