# Supplementary material for: Psychosocial burden and professional and social support in patients with hereditary transthyretin amyloidosis (ATTRv) and their relatives in Italy
Source: Orphanet J Rare Dis. 2021 Apr 7;16:163. doi: 10.1186/s13023-021-01812-6 (PMC8028211; doi:10.1186/s13023-021-01812-6)
Supplement: Supplementary file 1 — Additional file 1. Table S1, entitled “Help in daily activities needed by the patients with ATTRv and provided by their relatives in the past two months”; Table S2, entitled “Practical and psychological burden experienced by patients with ATTRv (N=141) in the last two months”; Table S3, entitled “ Perceived support received from professionals and social network by patients with ATTRv (N=141)”; Table S4, entitled “Practical and psychological burden experienced by relatives of patients with ATTRv (N=69) in the last two months”; Table S5, entitled “Perceived support received from professionals and social network by relatives of patients with ATTRv (N=69)”. [file 13023_2021_1812_MOESM1_ESM.docx]

.

| **Table S1.**  *Help in daily activities needed by the patients with ATTRv and provided by their relatives in the past two months.* | | | | | | | | |
| --- | --- | --- | --- | --- | --- | --- | --- | --- |
|  | Never | | Sometimes | | Often | | Always | |
|  | N | % | N | % | N | % | N | % |
| Help needed by the patients (N=141) |  |  |  |  |  |  |  |  |
| In getting around | 70 | 49.6 | 26 | 18.4 | 9 | 6.4 | 36 | 25.5 |
| In feeding oneself | 110 | 78.0 | 14 | 9.9 | 8 | 5.7 | 9 | 6.4 |
| In washing oneself | 93 | 66.0 | 13 | 9.2 | 11 | 7.8 | 24 | 17.0 |
| In dressing oneself | 83 | 58.9 | 19 | 13.5 | 12 | 8.5 | 27 | 19.1 |
| In going to visit and taking medications | 78 | 55.3 | 16 | 11.3 | 9 | 6.4 | 38 | 27.0 |
| Subscale’s mean scores ± SD | 1.8 ± .1 | | | | | | | |
| Help provided by the relatives (N=69) |  |  |  |  |  |  |  |  |
| In getting around | 28 | 40.6 | 10 | 14.5 | 14 | 20.3 | 17 | 24.6 |
| In feeding oneself | 49 | 71.0 | 8 | 11.6 | 6 | 8.7 | 6 | 8.7 |
| In washing oneself | 37 | 53.6 | 10 | 14.5 | 6 | 8.7 | 16 | 23.2 |
| In dressing oneself | 34 | 49.3 | 16 | 23.2 | 4 | 5.8 | 15 | 21.7 |
| In going to visit and taking medications | 27 | 39.1 | 12 | 17.4 | 6 | 8.7 | 24 | 34.8 |
| Subscale’s mean scores ± SD | 2.0 ± 1.1 | | | | | | | |

| **Table S2.**  *Practical and psychological burden experienced by patients with ATTRv (N=141) in the last two months.* | | | | | | | | |
| --- | --- | --- | --- | --- | --- | --- | --- | --- |
|  | Never | | Sometimes | | Often | | Always | |
|  | N | % | N | % | N | % | N | % |
| Practical burden |  |  |  |  |  |  |  |  |
| I have had to wake up during the night | 43 | 30.9 | 58 | 41.7 | 25 | 18.0 | 13 | 9.4 |
| I have had to neglect my hobbies and things I like doing in my free time | 38 | 27.1 | 26 | 18.6 | 43 | 30.7 | 33 | 23.6 |
| I have had difficulty in going on Sunday outings | 40 | 33.3 | 21 | 17.5 | 28 | 23.3 | 31 | 25.8 |
| I found it difficult to have friends at home | 64 | 45.7 | 42 | 29.8 | 21 | 14.9 | 14 | 9.9 |
| I found it difficult to meet friends and people I like to spend my leisure time | 64 | 45.7 | 40 | 28.6 | 29 | 20.7 | 7 | 5.0 |
| I found it difficult to carry out my usual work or household activities | 45 | 32.6 | 32 | 23.2 | 23 | 16.7 | 38 | 27.5 |
| I had to neglect other family members | 67 | 51.5 | 28 | 21.5 | 26 | 20.0 | 9 | 6.9 |
| I had difficulty in going on holiday | 45 | 49.9 | 19 | 17.3 | 14 | 12.7 | 32 | 29.1 |
| I had economic difficulties | 100 | 72.5 | 30 | 21.7 | 5 | 3.6 | 3 | 2.2 |
| Subscale’s mean scores ± SD | 2.1 ± .7 | | | | | | | |
| Psychological burden |  |  |  |  |  |  |  |  |
| I felt that I would not be able to bear this situation much longer | 63 | 44.7 | 51 | 36.2 | 25 | 17.7 | 2 | 1.4 |
| I cried or felt depressed | 57 | 40.4 | 68 | 48.2 | 16 | 11.3 | 0 | 0 |
| I worry for the future of other family members | 27 | 19.4 | 51 | 36.7 | 30 | 21.6 | 31 | 22.3 |
| When I went to a public place with my ill relative, I felt that everyone was watching us | 88 | 69.3 | 23 | 18.1 | 8 | 6.3 | 8 | 6.3 |
| I feel guilty because I believe that I or my spouse may have passed on the illness to our relative | 46 | 41.8 | 28 | 25.5 | 17 | 15.5 | 19 | 17.3 |
| If I did not have this problem, everything would be all right in the family | 55 | 39.6 | 30 | 21.6 | 21 | 15.1 | 33 | 23.7 |
| When I think of how I was beforehand and how I am now, I feel disappointed | 31 | 22.3 | 45 | 32.4 | 34 | 24.5 | 29 | 20.9 |
| If there were no family, there would be no one to take care of me. | 48 | 34.0 | 33 | 23.4 | 26 | 18.4 | 34 | 24.1 |
| Subscale’s mean scores ± SD | 2.1 ± .7 | | | | | | | |

| **Table S3.**  *Perceived support received from professionals and social network by patients with ATTRv (N=141).* | | | | | | | | |
| --- | --- | --- | --- | --- | --- | --- | --- | --- |
|  | N | % | N | % | N | % | N | % |
| Professional support | None | | Hardly any | | Some, but not enough | | Yes, adequately | |
| I have received information from doctors on my illness | 5 | 3.6 | 1 | .7 | 11 | 7.9 | 123 | 87.9 |
| I have received information from doctors on my illness treatments | 5 | 3.6 | 1 | .7 | 14 | 10.1 | 119 | 85.6 |
| I have received information from doctors on what to do in emergencies | 24 | 17.9 | 12 | 9.0 | 20 | 14.9 | 78 | 58.2 |
|  | Not at all | | A little | | Quite a lot | | Very much | |
| Doctors that are taking care of me, are helping me | 2 | 1.4 | 13 | 9.2 | 53 | 37.6 | 73 | 51.8 |
|  | Not at all | | A little | | Quite confident | | Completely confident | |
| In emergencies, I am confident that help will be provided immediately by professionals | 1 | .7 | 9 | 6.4 | 68 | 48.6 | 62 | 44.3 |
| Subscale’s mean scores ± SD | 3.5 ± .5 | | | | | | | |
| Social network support in emergencies | Not at all | | A little | | Quite confident | | Completely confident | |
| In an emergency, I am confident that help will be provided by my relatives or friends | 3 | 2.1 | 6 | 4.3 | 35 | 25.0 | 96 | 68.6 |
|  | Not at all | | I can ask just 1 person | | I can ask 2-3 persons | | I can ask more than 3 persons | |
| When things are going particularly badly, I can ask relatives or friends to help or support our family | 9 | 6.5 | 13 | 9.4 | 62 | 44.6 | 65 | 39.6 |
| Subscale’s mean scores ± SD | 3.4 ± .7 | | | | | | | |

| **Table S4.**  *Practical and psychological burden experienced by relatives of patients with ATTRv (N=69) in the last two months.* | | | | | | | | |
| --- | --- | --- | --- | --- | --- | --- | --- | --- |
|  | Never | | Sometimes | | Often | | Always | |
|  | N | % | N | % | N | % | N | % |
| Practical burden |  |  |  |  |  |  |  |  |
| I have had to wake up during the night | 29 | 42.0 | 25 | 36.2 | 11 | 15.9 | 4 | 5.8 |
| I have had to neglect my hobbies and things I like doing in my free time | 27 | 39.1 | 21 | 30.4 | 11 | 15.9 | 10 | 14.5 |
| I have had difficulty in going on Sunday outings | 24 | 39.3 | 15 | 24.6 | 6 | 9.8 | 16 | 26.2 |
| I found it difficult to have friends at home | 34 | 49.3 | 20 | 29.0 | 10 | 14.5 | 5 | 7.2 |
| I found it difficult to meet friends and people I like to spend my leisure time | 31 | 44.9 | 21 | 30.4 | 13 | 18.8 | 4 | 5.8 |
| I found it difficult to carry out my usual work or household activities | 33 | 47.8 | 27 | 39.1 | 5 | 7.2 | 4 | 5.8 |
| I had to neglect other family members | 38 | 56.7 | 19 | 28.4 | 7 | 10.4 | 3 | 4.5 |
| I had difficulty in going on holiday | 22 | 40.7 | 10 | 18.5 | 9 | 16.7 | 13 | 24.1 |
| I had economic difficulties | 52 | 76.5 | 11 | 16.2 | 1 | 1.5 | 4 | 5.9 |
| Subscale’s mean scores ± SD | 1.9 ± .7 | | | | | | | |
| Psychological burden |  |  |  |  |  |  |  |  |
| I felt that I would not be able to bear this situation much longer | 34 | 49.3 | 28 | 40.6 | 3 | 4.3 | 4 | 5.8 |
| I cried or felt depressed | 28 | 40.6 | 27 | 39.1 | 10 | 14.5 | 4 | 5.8 |
| I worry for the future of other family members | 17 | 25.0 | 26 | 38.2 | 16 | 23.5 | 9 | 13.2 |
| When I went to a public place with my ill relative, I felt that everyone was watching us | 44 | 69.8 | 12 | 19.0 | 4 | 6.3 | 3 | 4.8 |
| I feel guilty because I believe that I or my spouse may have passed on the illness to our relative | 23 | 60.5 | 9 | 23.7 | 2 | 5.3 | 4 | 10.5 |
| If he/she did not have this problem, everything would be all right in the family | 35 | 51.5 | 13 | 19.1 | 8 | 11.8 | 12 | 17.6 |
| When I think of how our ill relative was beforehand and how he/she is now, I feel disappointed | 15 | 22.1 | 14 | 20.6 | 15 | 22.1 | 24 | 35.3 |
| If there were no family, there would be no one to take care of him/her. | 24 | 35.3 | 11 | 16.2 | 13 | 19.1 | 20 | 29.4 |
| Subscale’s mean scores ± SD | 2.0 ± .7 | | | | | | | |

| **Table S5.**  *Perceived support received from professionals and social network by relatives of patients with ATTRv (N=69).* | | | | | | | | |
| --- | --- | --- | --- | --- | --- | --- | --- | --- |
|  | N | % | N | % | N | % | N | % |
| Professional support | None | | Hardly any | | Some, but not enough | | Yes, adequately | |
| I have received information from doctors on my ill relative’s illness | 7 | 10.3 | 3 | 4.4 | 9 | 13.2 | 49 | 72.1 |
| I have received information from doctors on my ill relative’s treatments | 7 | 10.3 | 4 | 5.9 | 4 | 5.9 | 53 | 77.9 |
| I have received information from doctors on what to do in emergencies concerning my ill relative | 20 | 29.9 | 8 | 11.9 | 11 | 16.4 | 28 | 41.8 |
|  | Not at all | | A little | | Quite a lot | | Very much | |
| Doctors that are taking care of my ill relative, are helping him/her | 2 | 2.9 | 8 | 11.6 | 28 | 40.6 | 31 | 44.9 |
|  | Not at all | | A little | | Quite confident | | Completely confident | |
| In an emergency concerning my ill relative, I am confident that help will be provided immediately by professionals | 0 | 0 | 5 | 7.2 | 38 | 55.1 | 26 | 37.7 |
| Subscale’s mean scores ± SD | 3.5 ± .5 | | | | | | | |
| Social network support in emergencies | Not at all | | A little | | Quite confident | | Completely confident | |
| In an emergency concerning my ill relative, I am confident that help will be provided by my relatives or friends | 5 | 7.2 | 6 | 8.7 | 26 | 37.7 | 32 | 46.4 |
|  | Not at all | | I can ask just 1 person | | I can ask 2-3 persons | | I can ask more than 3 persons | |
| When things are going particularly badly, I can ask relatives or friends to help or support our family | 7 | 10.1 | 9 | 13.0 | 29 | 42.0 | 24 | 34.8 |
| Subscale’s mean scores ± SD | 3.4 ± .7 | | | | | | | |
